# Supplementary material for: Transgenic expression of cif genes from Wolbachia strain wAlbB recapitulates cytoplasmic incompatibility in Aedes aegypti
Source: Nat Commun. 2024 Jan 29;15:869. doi: 10.1038/s41467-024-45238-7 (PMC10825118; doi:10.1038/s41467-024-45238-7)
Supplement: Supplementary file 1 — Supplementary Information [file 41467_2024_45238_MOESM1_ESM.pdf]

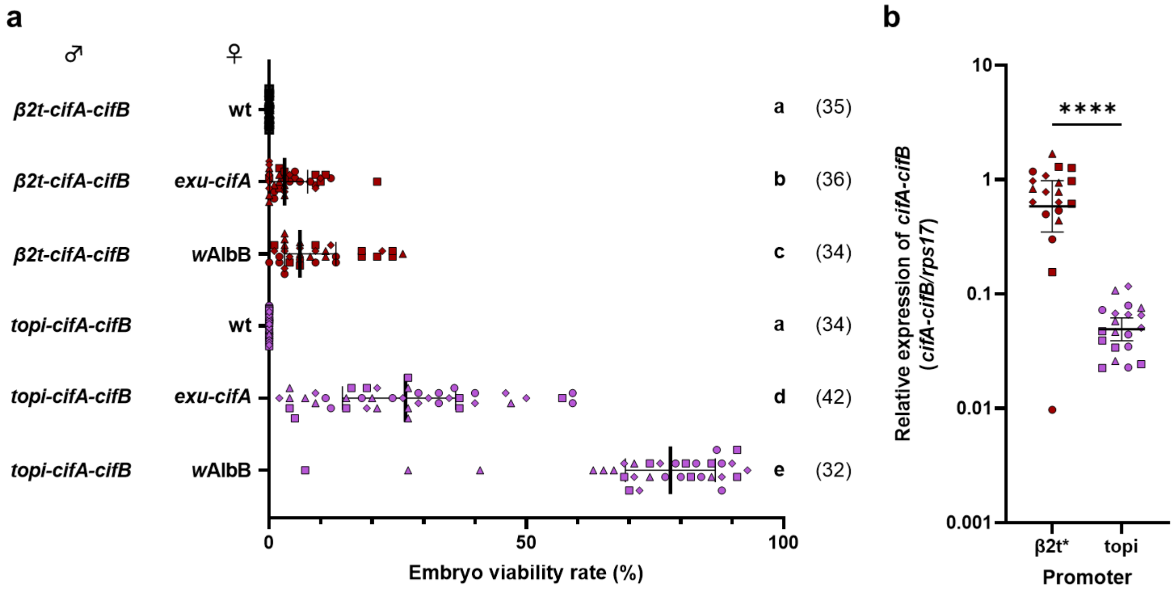

Supplementary Fig. 1 | Results of incompatibility crosses were consistent between different genomic insertion lines. a, Lowering the dosage of *cifA-cifB* increases the rescue capability of either transgenic (*exu*-cifA) or *w*AlbB-carrying females. Lines denote median and error bars interquartile ranges, numbers in parentheses denote the *n*. Letters indicate significant differences with an α=0.05 calculated by a Kruskal-Wallis test followed by a two-stage linear step-up procedure of Benjamini, Krieger, and Yekutieli to correct for multiple comparisons, individual P-values are listed in Source Data File. **b,** The relative expression of *cifA-cifB* was higher when controlled by the *β2t* as opposed to the *topi* promoter (unpaired t-test p<0.0001, mean and s.d. are shown, the *n*=20 for both groups). In both **a,** and **b,** circle, square, triangle and diamond symbols represent biological replicates from *β2t-cifA-cifB* and *topi-cifA-cifB* insertion sites 1,2,3, and 4 respectively. Source data are provided as a Source Data file.

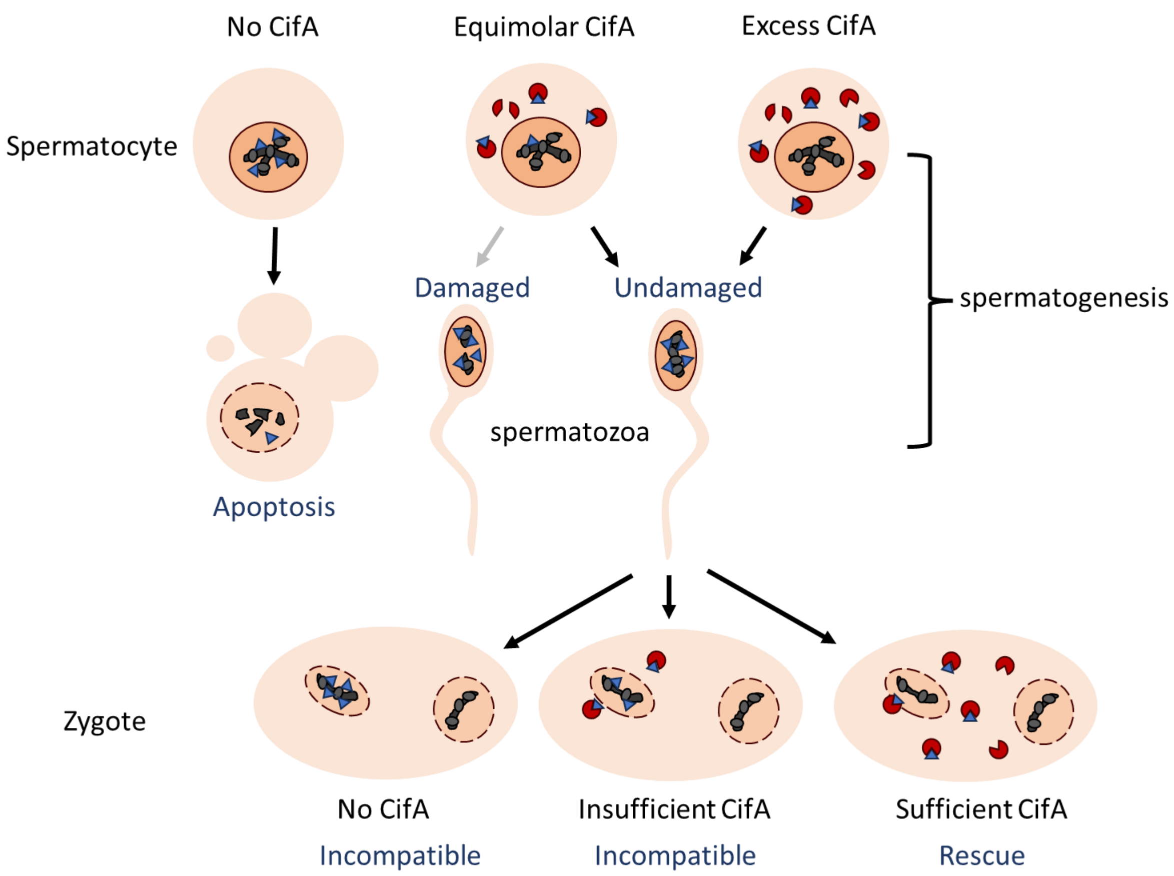

Supplementary Fig. 2 | Schematic of hypothesised interpretation of results. During spermatogenesis in the absence of CifA (red circular sector), CifB (blue triangle) may bind to DNA and the resulting toxicity leads to the premature termination of spermatogenesis (*β2t-cifB*). When CifA is present, CifA:CifB heterodimer formation prevents CifB from binding directly to the DNA. Equimolar expression of *cifA* and *cifB* (*cifA*-T2A-cifB) can stochastically lead to some unbound CifB, which can result in DNA damage depending on the amount of active CifB present [expression of both *cif* genes under the *topi* promoter does not result in damage, but a higher level of expression (*β2t*) results in some damage]. Excess *cifA* expression (*β2t-cifA; β2t-cifA-cifB*) reduces the chance of unbound CifB binding to the DNA, therefore preventing toxicity. CifB localises to spermatozoa nuclei[1-3], whereas CifA appears to degrade (broken red circular sector) and is not loaded into sperm nuclei[1,2]. Probably due to the tight compaction of DNA in spermatozoa nuclei, loaded CifB does not result in toxicity, however, upon chromatin decondensation after fertilisation the CifB toxin becomes active. If there is no CifA (e.g. wild-type) in the zygote, or the level of deposited CifA is insufficient to counteract the level of paternally deposited CifB, then embryonic lethality occurs. This lethality is rescued if the level of maternally deposited CifA is sufficient. Due to higher rates of CifA deposition there is less lethality in the offspring of *shu-cifA* females in comparison to *exu-cifA*. Damaged spermatozoa will always result in inviable offspring regardless of the levels of CifA in the zygote.

Supplementary Fig. 2 references

Horard B, Terretaz K, Gosselin-Grenet A-S, Sobry H, Sicard M, Landmann F *et al.* Paternal transmission of the *Wolbachia* CidB toxin underlies cytoplasmic incompatibility. *Current Biology* 2022; **32**: 1319-1331.e5.

Terretaz K, Horard B, Weill M, Loppin B, Landmann F. Functional analysis of *Wolbachia* Cid effectors unravels cooperative interactions to target host chromatin during replication. *PLoS Pathog* 2023; **19**: e1011211.

Kaur R, Leigh B, Ritchie I, Bordenstein S. The Cif proteins from *Wolbachia* prophage WO modify sperm genome integrity to establish cytoplasmic incompatibility. *PLOS Biology* 2022; 20(5): e3001584,
