# Supplementary material for: Transgenic expression of cif genes from Wolbachia strain wAlbB recapitulates cytoplasmic incompatibility in Aedes aegypti
Source: Nat Commun. 2024 Jan 29;15:869. doi: 10.1038/s41467-024-45238-7 (PMC10825118; doi:10.1038/s41467-024-45238-7)
Supplement: Supplementary file 3 — Description of Additional Supplementary Files [file 41467_2024_45238_MOESM3_ESM.pdf]

Description of Additional Supplementary Files

Supplementary Movie 1. Timelapse of wild-type and *β2t-cifB* male reproductive tissues. Mature spermatozoa were released (black arrow) from squished reproductive tissues dissected from wild-type (WT) males but not *β2t-cifB* males, tissues were imaged at 10x magnification.
