# Supplementary material for: Transgenic expression of cif genes from Wolbachia strain wAlbB recapitulates cytoplasmic incompatibility in Aedes aegypti
Source: Nat Commun. 2024 Jan 29;15:869. doi: 10.1038/s41467-024-45238-7 (PMC10825118; doi:10.1038/s41467-024-45238-7)
Supplement: Supplementary file 5 — Reporting Summary [file 41467_2024_45238_MOESM5_ESM.pdf]

nature portfolio

Corresponding author(s): Steven Sinkins

Last updated by author(s): Oct 3, 2023

Reporting Summary

Nature Portfolio wishes to improve the reproducibility of the work that we publish. This form provides structure for consistency and transparency in reporting. For further information on Nature Portfolio policies, see our Editorial Policies and the Editorial Policy Checklist.

Statistics

For all statistical analyses, confirm that the following items are present in the figure legend, table legend, main text, or Methods section.

n/a	Confirmed	
☐	☒	The exact sample size (*n*) for each experimental group/condition, given as a discrete number and unit of measurement
☐	☒	A statement on whether measurements were taken from distinct samples or whether the same sample was measured repeatedly
☐	☒	The statistical test(s) used AND whether they are one- or two-sided<br>*Only common tests should be described solely by name; describe more complex techniques in the Methods section.*
☐	☒	A description of all covariates tested
☐	☒	A description of any assumptions or corrections, such as tests of normality and adjustment for multiple comparisons
☐	☒	A full description of the statistical parameters including central tendency (e.g. means) or other basic estimates (e.g. regression coefficient) AND variation (e.g. standard deviation) or associated estimates of uncertainty (e.g. confidence intervals)
☐	☒	For null hypothesis testing, the test statistic (e.g. *F*, *t*, *r*) with confidence intervals, effect sizes, degrees of freedom and *P* value noted<br>*Give P values as exact values whenever suitable.*
☒	☐	For Bayesian analysis, information on the choice of priors and Markov chain Monte Carlo settings
☒	☐	For hierarchical and complex designs, identification of the appropriate level for tests and full reporting of outcomes
☒	☐	Estimates of effect sizes (e.g. Cohen's *d*, Pearson's *r*), indicating how they were calculated

Our web collection on statistics for biologists contains articles on many of the points above.

Software and code

Policy information about availability of computer code

Data collection	Confocal images were acquired using the Zeiss Zen black 2.3 SP1 software, and RT-qPCR experiments were conducted using the QuantStudio Design & Analysis Software v1.5.1.
Data analysis	GraphPad Prism 9.2.0

For manuscripts utilizing custom algorithms or software that are central to the research but not yet described in published literature, software must be made available to editors and reviewers. We strongly encourage code deposition in a community repository (e.g. GitHub). See the Nature Portfolio guidelines for submitting code & software for further information.

Data

Policy information about availability of data

All manuscripts must include a data availability statement. This statement should provide the following information, where applicable:

- Accession codes, unique identifiers, or web links for publicly available datasets
- A description of any restrictions on data availability
- For clinical datasets or third party data, please ensure that the statement adheres to our policy

Source data are provided with this paper. The source data, statistical analysis tests, and their outcomes used for figures are available to view in the Source Data file. The plasmid DNA sequences used to generate transgenic lines in this study are deposited in GenBank with the accession codes OR961086, OR961087, OR961088, OR961089, OR961090, and OR961091.

Research involving human participants, their data, or biological material

Policy information about studies with human participants or human data. See also policy information about sex, gender (identity/presentation), and sexual orientation and race, ethnicity and racism.

Reporting on sex and gender	The human blood was a surplus obtained from the Scottish National Blood Transfusion service, this service was anonymous and therefore donor information was unavailable.
Reporting on race, ethnicity, or other socially relevant groupings	The human blood was a surplus obtained from the Scottish National Blood Transfusion service, this service was anonymous and therefore donor information was unavailable.
Population characteristics	The human blood was a surplus obtained from the Scottish National Blood Transfusion service, this service was anonymous and therefore donor information was unavailable.
Recruitment	The human blood was a surplus obtained from the Scottish National Blood Transfusion service, this service was anonymous and therefore donor information was unavailable.
Ethics oversight	The use of human blood does not require a research ethics committee approval since the blood was used solely for feeding mosquitoes and then disposed after use. No genetic testing or manipulation, assay development or diagnostic research was involved and the blood was excess from the Scottish National Blood Transfusion Service (SNTBS) which would have otherwise been disposed of. The use of blood in this manner was approved by the SNBTS Committee for the Governance of Blood and Tissue Samples for Non-therapeutic Use, and Donor Research.

Note that full information on the approval of the study protocol must also be provided in the manuscript.

Field-specific reporting

Please select the one below that is the best fit for your research. If you are not sure, read the appropriate sections before making your selection.

☒ Life sciences ☐ Behavioural & social sciences ☐ Ecological, evolutionary & environmental sciences

For a reference copy of the document with all sections, see nature.com/documents/nr-reporting-summary-flat.pdf

Life sciences study design

All studies must disclose on these points even when the disclosure is negative.

Sample size	Due to constraints inherent in crossing, individualizing, and obtaining egg batches from female mosquitoes, sample sizes were necessarily small. This was particularly true for crosses that required the generation and crossing of females carrying two independent genomic transgene insertions.<br>For crosses with cifA;cifB double heterozygotes the minimum total sample size for a comparison was 26 individuals (12 and 14 for each group). This resulted in a power of 0.33 to detect an effect size of 50% with an alpha prob of 0.05% (non-centrality = 1.242, critical-t = 1.714). However, this was sufficient to detect a significant effect in CI rescue of an additional CifA insertion.<br>For all other crosses (involving individuals with a single transgene insertion) the minimum number of egg batches was a total of 33 (4 (22 and 22 for each group), giving a power estimate of 0.48 to detect an effect size of 50% with an alpha prob of 0.05% (non-centrality = 1.62, critical-t = 1.68) - however, many comparisons used far larger samples than these.<br>It should be noted that the expected effect size was far higher than 50% in many cases. In experiments involving CI induction the effect was often a reduction in mean egg hatch from ~70% (control) to 0% (treatment). Similarly, for experiments concerning CI rescue the increase in egg hatch rate was from 0% (control) to ~50-60% (treatment).
Data exclusions	No data was excluded.
Replication	Crosses were performed twice with independent mosquito generations to ensure that results were repeatable. The observed trends were consistent between independent trials.
Randomization	Mosquito colonies were all reared under standardized conditions, resulting in highly homogeneous populations. Mosquitoes used in crosses were selected at random from these populations with no selection criteria except the presence/absence of transgenes.
Blinding	Experiments involved the simple counting of mosquito eggs (either developed or undeveloped) and hatched larvae. As the nature of this counting procedure does not present an opportunity for unconscious bias, no blinding of groups was performed. Moreover, the work was performed and confirmed by several different researchers.

Reporting for specific materials, systems and methods

We require information from authors about some types of materials, experimental systems and methods used in many studies. Here, indicate whether each material, system or method listed is relevant to your study. If you are not sure if a list item applies to your research, read the appropriate section before selecting a response.

Materials & experimental systems

n/a	Involved in the study
☒	☐ Antibodies
☒	☐ Eukaryotic cell lines
☒	☐ Palaeontology and archaeology
☒	☐ Animals and other organisms
☒	☐ Clinical data
☒	☐ Dual use research of concern
☒	☐ Plants

Methods

n/a	Involved in the study
☒	☐ ChIP-seq
☒	☐ Flow cytometry
☒	☐ MRI-based neuroimaging
